# Duplication and Divergence of Leucine-Rich Repeat Receptor-Like Protein Kinase (*LRR-RLK*) Genes in Basal Angiosperm *Amborella trichopoda*

**DOI:** 10.3389/fpls.2016.01952

**Published:** 2016-12-23

**Authors:** Ping-Li Liu, Lu-Lu Xie, Peng-Wei Li, Jian-Feng Mao, Hui Liu, Shu-Min Gao, Peng-Hao Shi, Jun-Qing Gong

**Affiliations:** ^1^College of Biological Sciences and Biotechnology, Beijing Forestry UniversityBeijing, China; ^2^Department of Chinese Cabbage, Institute of Vegetables and Flowers, Chinese Academy of Agricultural SciencesBeijing, China; ^3^State Key Laboratory of Systematic and Evolutionary Botany, Institute of Botany, Chinese Academy of SciencesBeijing, China

**Keywords:** *Amborella trichopoda*, leucine-rich repeat receptor-like kinase (LRR-RLK), functional divergence, protein structure, protein motif, gene structure, expression

## Abstract

Leucine-rich repeat receptor-like protein kinases (LRR-RLKs) are the largest group of receptor-like kinases, which are one of the largest protein superfamilies in plants, and play crucial roles in development and stress responses. Although the evolution of *LRR-RLK* families has been investigated in some eudicot and monocot plants, no comprehensive evolutionary studies have been performed for these genes in basal angiosperms like *Amborella trichopoda*. In this study, we identified 94 *LRR-RLK* genes in the genome of *A. trichopoda*. The number of *LRR-RLK* genes in the genome of *A. trichopoda* is only 17–50% of that of several eudicot and monocot species. Tandem duplication and whole-genome duplication have made limited contributions to the expansion of *LRR-RLK* genes in *A. trichopoda*. According to the phylogenetic analysis, all *A. trichopoda LRR-RLK* genes can be organized into 18 subfamilies, which roughly correspond to the *LRR-RLK* subfamilies defined in *Arabidopsis thaliana*. Most *LRR-RLK* subfamilies are characterized by highly conserved protein structures, motif compositions, and gene structures. The unique gene structure, protein structures, and protein motif compositions of each subfamily provide evidence for functional divergence among LRR-RLK subfamilies. Moreover, the expression data of *LRR-RLK* genes provided further evidence for the functional diversification of them. In addition, selection analyses showed that most LRR-RLK protein sites are subject to purifying selection. Our results contribute to a better understanding of the evolution of LRR-RLK gene family in angiosperm and provide a framework for further functional investigation on *A. trichopoda* LRR-RLKs.

## Introduction

All living organisms sense and conduct signals through cell surface receptors. In plants, cellular signal transduction is mainly mediated by receptor-like kinases (RLKs), a protein superfamily. RLKs contain three functional domains: a ligand-binding extracellular domain, a membrane-spanning domain, and an intracellular serine/threonine kinase domain (Shiu and Bleecker, 2001). The extracellular domains of RLK proteins are highly divergent. Based on the structure of the extracellular domain and phylogenetic analysis of the kinase domains (KDs), RLK proteins of *Arabidopsis thaliana* were divided into more than 50 families. The largest group is the leucine-rich repeat RLK family (LRR-RLK).

LRR-RLK proteins are receptor-like kinases that contain leucine-rich repeats (LRRs) in their extracellular domain (Shiu and Bleecker, 2001). The LRR is a widespread structural motif of 20–30 amino acids with conserved leucines, which build the domain from tandem repeats (Torii, 2004). The LRR domains of LRR-RLK proteins usually vary in number and in the distribution pattern of LRR repeats, and LRR diversity enables LRR-RLKs to sense a variety of ligands, including small molecules, peptides, and entire proteins (Bojar et al., 2014). The kinase domains of LRR-RLK proteins are common in protein kinases. It contains 12 conserved subdomains that fold into a similar three-dimensional catalytic core with a two-lobed structure (Hanks et al., 1988; Hanks and Hunter, 1995). The small lobe includes subdomains I–IV, whereas the large lobe includes subdomains VIA–XI. Kinase domains catalyze phosphotransfer according to a common mechanism: the smaller lobe is primarily involved in anchoring and orienting the nucleotide, whereas the larger lobe is largely responsible for binding the peptide substrate and initiating phosphotransfer (Hanks and Hunter, 1995).

Gene duplications, often followed by functional diversification, have repeatedly played an important role in providing the raw material for the evolution of the species. Gene duplication is very prominent in the evolution of the *LRR-RLK* gene family in plants (Lehti-Shiu et al., 2009; Lehti-Shiu and Shiu, 2012). In eudicots, such as *A. thaliana, Brassica rapa, Solanum lycopersicum* and *Populus trichocarpa*, 213, 303, 234, and 379 *LRR-RLK* genes, respectively, have been identified from the analysis of genome sequences (Shiu and Bleecker, 2001; Zan et al., 2013; Rameneni et al., 2015; Wei et al., 2015). Based on the sequence similarity and domain conservation, as many as 467 genes were identified in the *Glycine max* genome (Zhou et al., 2016). In monocot *Oryza sativa*, 309 *LRR-RLK* genes were found via genome-wide identification (Sun and Wang, 2011). A recent study showed that another monocot *Triticum aestivum* has the largest number of *LRR-RLK* genes (531) as far as we know (Shumayla et al., 2016). Tandem duplication and whole genome duplication are major mechanisms underlying expansion of the *LRR-RLK* family in these species (Shiu and Bleecker, 2001, 2003; Sun and Wang, 2011; Zan et al., 2013; Zhou et al., 2016). After duplication, duplicated genes often accumulate mutations that lead to functional divergence. The biological roles of only a small number of LRR-RLK proteins are understood. However, there is clear genetic evidence for functional diversification of LRR-RLK proteins (Zhang et al., 2006). For example, LRR-RLKs have been found to play important roles in meristematic growth (Clark et al., 1997), embryogenesis (Nodine et al., 2007, 2011), secondary growth (Agusti et al., 2011), polar pollen tube growth (Chang et al., 2013), pollen self-incompatibility (Muschietti et al., 1998), ABA and brassinosteroid signal transduction, and responses to environmental signals (Li and Chory, 1997; Osakabe et al., 2005). LRR-RLK proteins are known to function as regulators of the defense response to bacterial pathogens, necrotrophic fungi, and viruses (Gómez-Gómez and Boller, 2000; Fontes et al., 2004; Llorente et al., 2005). Some LRR-RLK proteins are functionally redundant in regulating some aspects of *A. thaliana* growth and development (Eyüeboglu et al., 2007; Albrecht et al., 2008). For example, SERK1 and SERK2 play functionally redundant roles in the process of male microsporogenesis. SERK1 acts redundantly with BAK1 in brassinosteroid signaling, whereas BAK1 acts redundantly with SERK4 in cell death control (Albrecht et al., 2008). The functional redundancy of LRR-RLK family members complicates studies of their functions.

Although the evolution of *LRR-RLK* genes has been well studied in some eudicot and monocot species, much less information has been reported about these genes in basal angiosperms such as *Amborella trichopoda*. *A. trichopoda* is the single living representative of the sister lineage to all other extant flowering plants (Angiosperm) (Albert et al., 2013). As a basal angiosperm, *A. trichopoda* can be studied as a means of understanding the evolution of many aspect of the angiosperm genome, including the evolution of genes and gene families (Albert et al., 2013). In this study, we performed genome-wide searches for *LRR-RLK* gene sequences in the *A. trichopoda* genome and performed phylogenetic analyses to understand the relationships among these genes. According to the phylogenetic analyses, *LRR-RLK* genes were classified into subfamilies. The protein structures, protein motifs, and gene structures of the identified LRR-RLK genes were used to provide evidence for classification of the genes into subfamilies and, more importantly, indicated functional diversification. Furthermore, the expression profiles of *LRR-RLK* genes provided further evidence for the functional diversification of them. Finally, selection analyses indicated that most *LRR-RLK* gene sites were under purifying selection. Our results reveal important information regarding the evolution of the *LRR-RLK* gene family in angiosperms and provide a framework for further investigation of the functions of *A. trichopoda* LRR-RLKs.

## Methods

### Identification of *LRR-RLK* genes

The kinase domain sequences of representative proteins from each *LRR-RLK* subfamily of *A. thaliana* were used as queries to conduct Blastp searches (*E*-value cutoff <1 × 10^−10^) against the *A. trichopoda* protein databases available on Phytozome v11.0 (Goodstein et al., 2012), yielding 438 hits. Next, we manually checked whether each gene contained LRR domains and one KD domain (PF00560 and PF00069). Genes in the *A. trichopoda* genome v1.0 annotated with Pfam domains (PF00560 and PF00069) were downloaded from Phytozome v11.0. Identical and defective sequences were identified and eliminated by manual inspection in BioEdit. Next, potential kinase sequences were analyzed with CDD (http://www.ncbi.nlm.nih.gov/Structure/cdd/wrpsb.cgi) (Marchler-Bauer et al., 2011) to further verify the presence of LRR and KD domains. The candidates were analyzed with TMHMM v. 2.0 (http://www.cbs.dtu.dk/services/TMHMM/) (Krogh et al., 2001) to confirm the presence of transmembrane domains (TMs). Only sequences that contained LRRs in the ECD, TMs, and a KD were considered as *LRR-RLK*s.

### Genome distribution of *LRR-RLK* genes

In *A. trichopoda*, genome sequences were only assembled into scaffolds (Albert et al., 2013). All *LRR-RLK* genes identified in this study were mapped onto their corresponding scaffolds based on the physical positions of them. First, Physical positions of all *LRR-RLK* genes and scaffolds lengths were obtained from the Phytozome database. Then, MapInspect software (http://mapinspect.software.informer.com/) was used to produce the schematic diagrams of physical locations of *LRR-RLK* genes in scaffolds. As previous literature, tandem duplication cluster in this study was defined as a region containing two or more genes within 200 kb (Zan et al., 2013; Zhou et al., 2016). Furthermore, tandem duplication genes should show close relationship in phylogenetic tree. The tandem duplication clusters were identified and highlight in the image (Table 1).

**Table 1:** **Subgroups of LRR-RLK proteins from ***A. trichopoda*****.

**Sub**.	**R (N_ATh_/N_ATR_)**	**Range of LRRs**	**Motif pattern**	**Range of Introns**	**Homologous Arabidopsis genes**
I	8.2 (41/5)	**3**, 4	M_c_, M3, M8, M11, M13	10, 11, 15	MEE39, ISO1, RHS6, FRK1
II	2.3 (14/6)	**3**, **4**	M_c_,M3, M9, M11, M13	**10**, 8	SERK1-2, BAK1, BKK1, SARK, ATNIK1-3
III	2.6 (41/16)	3~17, **5**, **6**	M_c_, M13	**1**, **2**, 3	PRK1-6, RLK, RUL1, TMKL1, PXC1, RKL1, SIRK1, IMK2-3
IV	3 (3/1)	**6**	M_c_, M3, M11	**3**	
V	4.5 (9/2)	6, 8	M_c_, M11, M13	**15**	SRF1, SRF3-8, SUB/SRF9
VI-1	1.7 (5/3)	7~9	M_c_,	**6**, 9	
VI-2	4 (4/1)	**4**	M_c_,	**11**	MRH1
VII-1	2 (2/1)	**23**	M_c_, M3, M11, M13	**0**	
VII-2	1.7 (5/3)	13~18	M_*c*_, M3, M11,	1, **2**	PXC2
VIII-1	2 (8/4)	7, **11**	M_c_, M3, M11, M13	**18**, 19	
VIII-2	4 (12/3)	4, **8**	M_c_, M3, M11,	16, 17, 22	
IX	1.3 (4/3)	8, 9, 11	M_c_, M3, M11, M13	0, **1**	BARK1, TMK1
X^*^	1.7 (15/9)	14~28	M_c_, M11, M6, M13	**0**, 1	BIR1, BRL1-3, PSKR1-2, EMS1
XI^*^	1.3 (32/24)	17~27	M_c_, M3, M8, M11, M13	**1**	PXY, BAM1-3, PEPR
XII	1 (7/7)	10~26	M_c_, M3, M11, M13	**1**	FLS2, EFR
XIII-1	1 (3/3)	3, **4**	M_c_, M3, M8, M11, M13	**12**	FEI1-2
XIII-2	3 (3/1)	**19**	M_c_, M3, M8, M11, M13	**26**	ERECTA, ERL1-2,
XV	1 (2/2)	15, 18	M_c_, M3, M11, M13	0, 1	

*R is the ratio of gene number from A. thaliana to that from A. trichopoda. **N**_ATh_ indicates gene number from A. thaliana, **N**_ATR_ indicates gene number from A. trichopoda. Mc represents M1, M2, M4, M5, M7, M10, M12, M14, and M15. Bold number in range of LRRs and range of intron indicate most members with this numbers of LRR repeats or introns*.

### *LRR-RLK* gene alignments and phylogenetic analysis

Two data sets were used for the phylogenetic analysis. One data set consisted of *LRR-RLK* sequences from *A. trichopoda* and was used to investigate the evolutionary relationships among the *LRR-RLK* genes of *A. trichopoda*. The second data set consisted of *LRR-RLK* sequences obtained in the present study and previously reported in *A. thaliana* (Shiu and Bleecker, 2001). The second data set was used to explore the phylogenetic relationships of the *A. trichopoda* LRR-RLK proteins in relation to the LRR-RLK proteins in *A. thaliana*. The sequences in each data set were aligned separately using muscle with default settings (Gap opening penalty, −2.9; Gap extend, 0; Hydrophobicity Multiplier, 1.2; Clustering method, UPGMB) (Edgar, 2004). For both datasets, only the amino acid sequences of the kinase domain were subjected to phylogenetic analysis because the alignments of other positions were ambiguous. Phylogenetic trees were constructed using the maximum likelihood (ML) method implemented in RAxML (Stamatakis et al., 2008). The best-fit amino acid substitution models (LG+G for both datasets) for ML analyses were selected by MEGA6 (Tamura et al., 2013). The starting tree was obtained with BioNJ. Parameter values were estimated from the data. Branch support was estimated from 1000 bootstrap replicates. The trees were rooted at the midpoint.

### Protein structure analyses

All LRR-RLK proteins contain LRR, TM, and KD domains. However, the number of LRRs varies among LRR-RLK proteins. In *A. thaliana*, the members of each subfamily usually have the same number of LRRs. To explore patterns in the number of LRRs in the identified LRR-RLK genes, we analyzed the genes with CDD (http://www.ncbi.nlm.nih.gov/Structure/cdd/wrpsb.cgi) (Marchler-Bauer et al., 2011) and drew the LRR, TM, and KD domains of each LRR-RLK protein with illustrator. Next, the protein structures were mapped to each protein in the phylogenetic tree.

The KD domain contained 12 subdomains, which usually included some conserved amino acids that play important roles in the activity and regulation of kinases. Although the KD domain is relatively well conserved, divergence was found. To elucidate the evolution of the KD domain, conserved motifs were identified with Multiple Expectation Maximization for Motif Elicitation (MEME) v.4.10.2. (http://meme-suite.org/tools/meme) (Bailey et al., 2009). MEME was executed in zoop (zero or one occurrence per sequence) mode. Parameters were set as follows: maximum number of motifs, 15; minimum motif width, 6; maximum motif width, 50; and default settings for all other parameters. Besides, considering there may exist domains other than LRR-TM-Kinase, all protein identified in this study were analyzed with Pfam (http://pfam.xfam.org/).

### Gene structure analysis

To study gene structure evolution, the intron/exon structures for each gene were mapped to their corresponding genes. Genomic sequences of the *A. trichopoda* v.1.0 annotation were downloaded from Phytozome, after which untranslated regions were removed. Coding sequences were also downloaded from Phytozome. The intron/exon structures were determined by comparing CDS with their corresponding genomic DNA sequences, and schematics were generated using the Gene Structure Display Server (GSDS) v. 2.0 (http://gsds.cbi.pku.edu.cn/) (Hu et al., 2015).

### Test for evolutionary selection pressure

Comparison between non-synonymous and synonymous substitution rates (ω = d_N_/d_S_) is an effective method for detecting positive or purifying selection on protein-coding genes (Yang and Bielawski, 2000). We used this approach to assess selective forces acting on *LRR-RLK* genes of five subfamilies (I, II, III, VIII-1, and XII) with high bootstrap support and sequence number greater than four. The ω value was estimated using the codeml program in the PAMLX software (Xu and Yang, 2013). The codon alignments used as input for codeml were created with DAMBE5 (Xia, 2013). Six site models (model = 0; NSsites = 0, 1, 2, 3, 7, 8) were used for these subfamilies. Nested models were compared using likelihood ratio tests (LRTs) of the log likelihood (InL). 2|ΔlnL| values between models and degrees of freedom were used in a chi-square test with a significance threshold of *P* < 0.01. The M0 model assumes the same ω for all branches and all sites, whereas M3 uses a general discrete distribution with three site classes. This pair of model was compared to test for variable selective pressure among sites. The nearly neutral model (M1) assumes sites with ω ≤ 1, whereas the positive selection model (M2) adds a third class of sites with ω > 1 to M1. The beta model (M7) assumes a beta distribution for the ratio over sites, whereas the beta&ω model (M8) adds an extra class of sites with ω > 1 to M7. These two pairs of nested models (M1a and M2a, M7 and M8) were compared to test for evidence of sites under positive selection.

### Expression profile of LRR-RLK genes

For LRR-RLK gene expression analysis, Illumina RNA-seq data from whole plant, apical meristem and young leaves (AMYL), and premeiotic female buds were download from the NCBI SRA database (The aceesion numbers were SRX1668558, SRX1668559, and SRX1668560, respectively). Reads were filtered to obtain high quality clean reads using trimmomatic v. 0.32 (Bolger et al., 2014). Then, the clean reads longer than or equal to 40 bp were mapped to LRR-RLK genes using bwa-mem v. 0.7.12 software (Li, 2013) with default parameters. FPKM (Fragments per kilobase per million) values were calculated using customized script to remove the library size and the fragmentation bias. A heat map of the LRR-RLK genes was generated using pheatmap package (Kolde, 2012) of R software (https://www.r-project.org/).

## Results

### Identification and genome distribution of *LRR-RLK* genes in *A. trichopoda*

**Figure 1:**
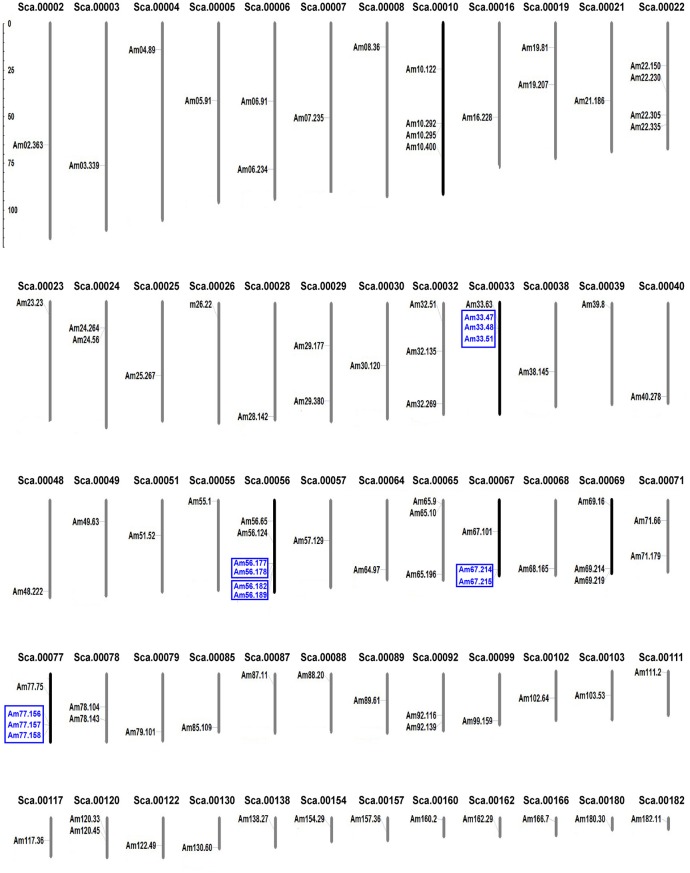
**Distribution of LRR-RLK genes on ***A. trichopoda*** scaffolds**. The scaffold numbers are given at the top of each scaffold, and genes probably derived from tandem duplication are highlight with blue and in blue boxes.

**Figure 2:**
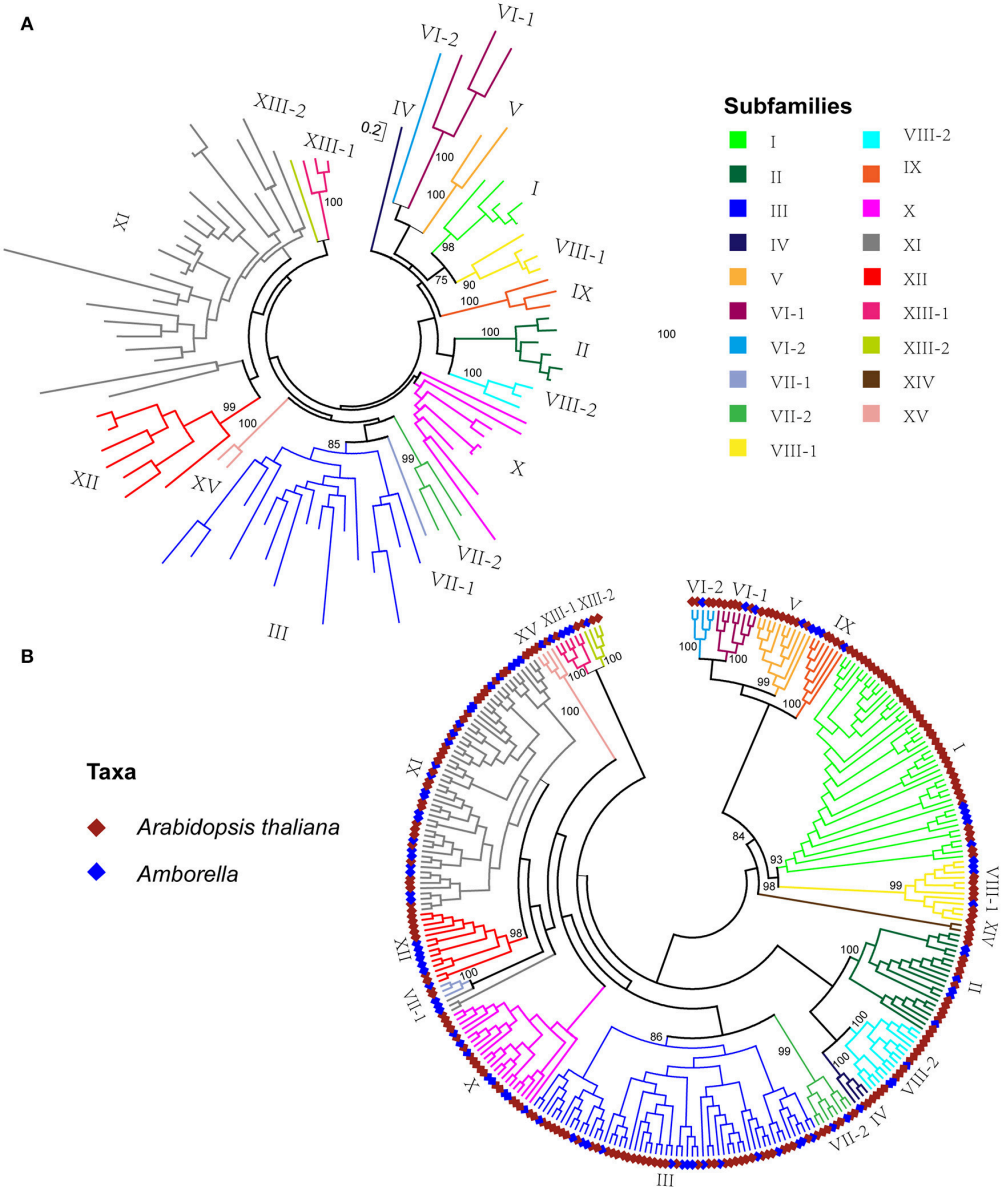
**Phylogenetic tree of LRR-RLK genes. (A)** Maximum likelihood tree of *LRR-RLK* genes in *Amborella trichopoda*. **(B)** Maximum likelihood tree of *LRR-RLK* genes in *Amborella trichopoda* and *Arabidopsis thaliana*. Bootstrap values of major clades are shown above branches. The full phylogeny is shown in Supplemental Figure 1.

In total, 94 *LRR-RLK* sequences were identified in the *A. trichopoda* genome. We renamed these genes and their corresponding full ID name in phytozome were in Supplemental Table 1. In *A. trichopoda*, genome sequences were only assembled into scaffolds (Albert et al., 2013). Physical positions of *LRR-RLK* genes obtained from the phytozome database were used to map them onto the corresponding scaffolds of *A. trichopoda*. Results showed that the 94 genes were located in 60 scaffolds (Figure 1). The numbers of genes in each scaffold varied from one to six. We grouped *LRR-RLK* genes into the same cluster if they were arranged in a genomic region with a maximum of 200 kb. In total, seven clusters were identified (Figure 1). One cluster contained three genes and the other clusters contained only two genes. Except one scaffold contained two cluster, all other scaffold contained one cluster (Figure 1). The tandem duplicated paralogs were eligible when they showed proximity in their chromosomal location (in the same cluster) and formed the same clade in the phylogenetic tree. Among the seven clusters, genes from two clusters were not included in the same clade (Figure 2). Hence, only five clusters (12 genes) could be taken as genes derived from tandem duplication, which represented about 12% (12/94) of *A. trichopoda LRR-RLK* genes.

### Phylogenetic analysis of *LRR-RLK* genes

The phylogenetic relationship of the *A. trichopoda LRR-RLK* sequences is shown in Figure 2A. In the ML tree, the sequences clearly fell into distinct clades, indicating that these natural groups can be assigned to different subfamilies. To better classify these subfamilies, the evolution of *A. trichopoda LRR-RLK* genes was evaluated through maximum-likelihood analysis incorporating well-described *LRR-RLK* sequences in the dicot *A. thaliana*. A previous study identified 213 *LRR-RLK* genes in the completely sequenced *A. thaliana* genome (Shiu and Bleecker, 2001, 2003). According to kinase-domain phylogeny, *A. thaliana LRR-RLK* genes can be classified into 15 subfamilies (Shiu and Bleecker, 2001, 2003). In this study, *A. thaliana LRR-RLK* genes resolved into broadly the same subfamilies in the phylogenetic trees (Figure 2B and Supplemental Figure 2) after adding *A. trichopoda* sequences. Therefore, we annotated these subfamilies using previously established nomenclature (Shiu and Bleecker, 2001), with a few modifications (Figure 2); for example, subfamilies VI, VII, and XIII were subdivided into subfamilies VI-1 and VI-2; VII-1 and VII-2, and XIII-1 and XIII-2, respectively. In total, *LRR-RLK* genes were classified into 19 subfamilies according to the phylogenetic tree (Figure 2B and Supplemental Figure 2). All subfamilies except subfamilies X and XI were supported as clades with moderate to high bootstrap support (>80). For subgroup X and XI, the topology varied between trees: either the group XI appears to be monophyletic clade with very low branch support or to be paraphyletic. As we could not confirm that it was monophyletic, they were labeled with an asterisk. Of the 19 *LRR-RLK* subfamilies (Figure 2), subfamily XIV did not include *LRR-RLK* genes from *A. trichopoda*. All other subfamilies included *LRR-RLK* genes from both *A. thaliana* and *A. trichopoda*. When *A. trichopoda LRR-RLK*s were clustered with *A. thaliana LRR-RLK*s (Figure 2B), the numbering for the *A. trichopoda LRR-RLK* subfamilies (Figure 2A) was determined based on the nomenclature of the majority of *A. thaliana* homologs within the same group. Hence, *LRR-RLK* genes *from A. trichopoda* were classified into 18 subfamilies. The number of genes between subfamilies was highly variable (Table 1), Subfamilies III and XI^*^ have the highest number of genes, with 16, 24 genes, respectively. The lowest numbers of genes are subfamilies IV, VI-2, VII-1, XIII-2, which only possessed one gene. XV also showed very low number of genes, with two genes. After comparison of the copy number of each subfamily between *A. trichopoda* and *A. thaliana*, we found that subfamilies I, VI-2, VIII-2, XIII-2 showed the largest expansion rate, with 8.2, 4, 4, 3, respectively. In most of other subfamilies, there were also more members in *A. thaliana* than in *A. thichopoda*. Among the homologous *A. thaliana* genes of *A. trichopoda LRR-RLK* genes in each subfamily, some are well studied. Those genes with known functions are list in Table 1.

Phylogenetic analysis of kinase domains enables delimitation of the major evolutionary lineages of the *LRR-RLK* subfamilies of *A. trichopoda*, but it provides little information about phylogenetic relationships between different subfamilies. As shown in the ML tree (Figure 2 and Supplemental Figures 1, 2), most deep nodes that represented the phylogenetic relationships between different *LRR-RLK* subfamilies had low support values, and they varied between trees constructed by ML analyses or NJ (not shown). This finding is similar to the results of a phylogenetic analysis of the kinase domains of *LRR-RLK* genes in other organisms (Sun and Wang, 2011) and is likely due to the fact that the kinase domain is relatively short and conserved, with relatively few informative character positions. Therefore, the inter-subfamily relationships shown in Figure 2 were omitted in the later discussion.

### Protein domain and motif analyses

**Figure 3:**
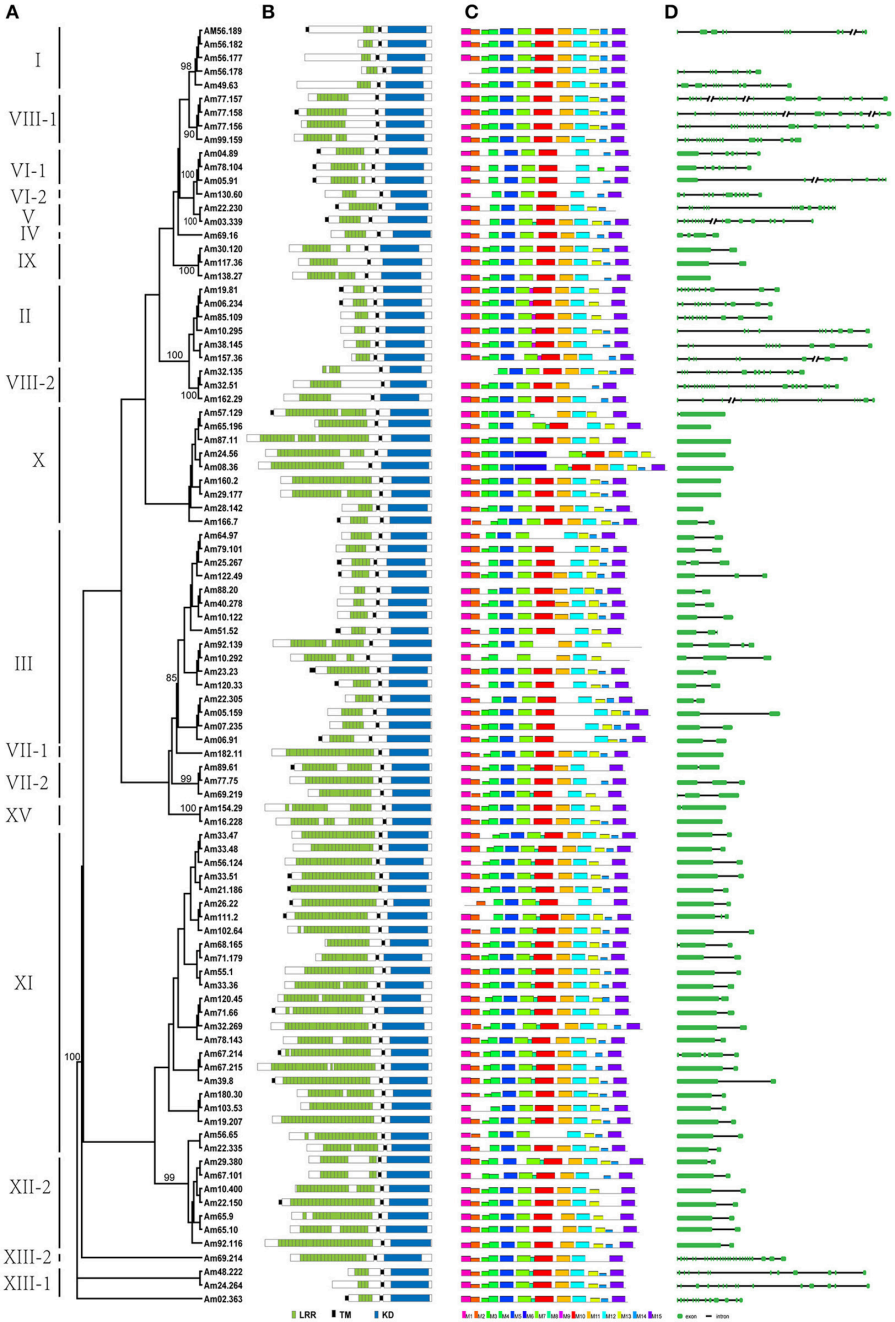
**ML tree of 94 LRR-RLK proteins from ***Amborella trichopoda***, with corresponding protein structure, motif, and gene structure. (A)** ML tree of 94 LRR-RLK proteins from *Amborella trichopoda*. The full names of each LRR-RLK protein are shown in Supplemental Table 1. The subfamily names are shown on the left. **(B)** Protein structures of LRR-RLK proteins. **(C)** MEME motif distribution of the kinase domain of each protein. **(D)** Gene structure of each LRR-RLK protein. The green boxes represent exons, lines represent introns, and each line with double slash indicates a long intron.

To investigate the protein structure characteristic of the LRR-RLK proteins in each subfamily, all LRR-RLK proteins of *A. trichopoda* were subjected to protein domain analyses in the CDD (http://www.ncbi.nlm.nih.gov/Structure/cdd/wrpsb.cgi). Protein structures were mapped to each protein in the phylogenetic tree (Figure 3) (Figure 3A is an unrooted cladgram presentation of the tree in Figure 2A). According to the protein structure analysis, members within each of the LRR-RLK subfamilies in *A. thaliana* tend to have a similar number of LRR repeats in the LRR domain, while members in different subfamilies have different numbers of LRR repeats in the LRR domain. As shown in Figures 3A,B and Table 1, this pattern can also be observed in the LRR-RLK proteins of *A. trichopoda*. For example, although 1 sequence in subfamily I has 4 LRR repeats in the LRR domain, the other 4 members of this subfamily have 3 LRR repeats in that domain. All members of subfamily II have 3 or 4 LRR repeats in the LRR domain. Three of the four members of subfamily VIII-1 have 11 LRR repeats in the LRR domain. The members of subfamily XII have 16–23 LRR repeats in the LRR domain.

**Figure 4:**
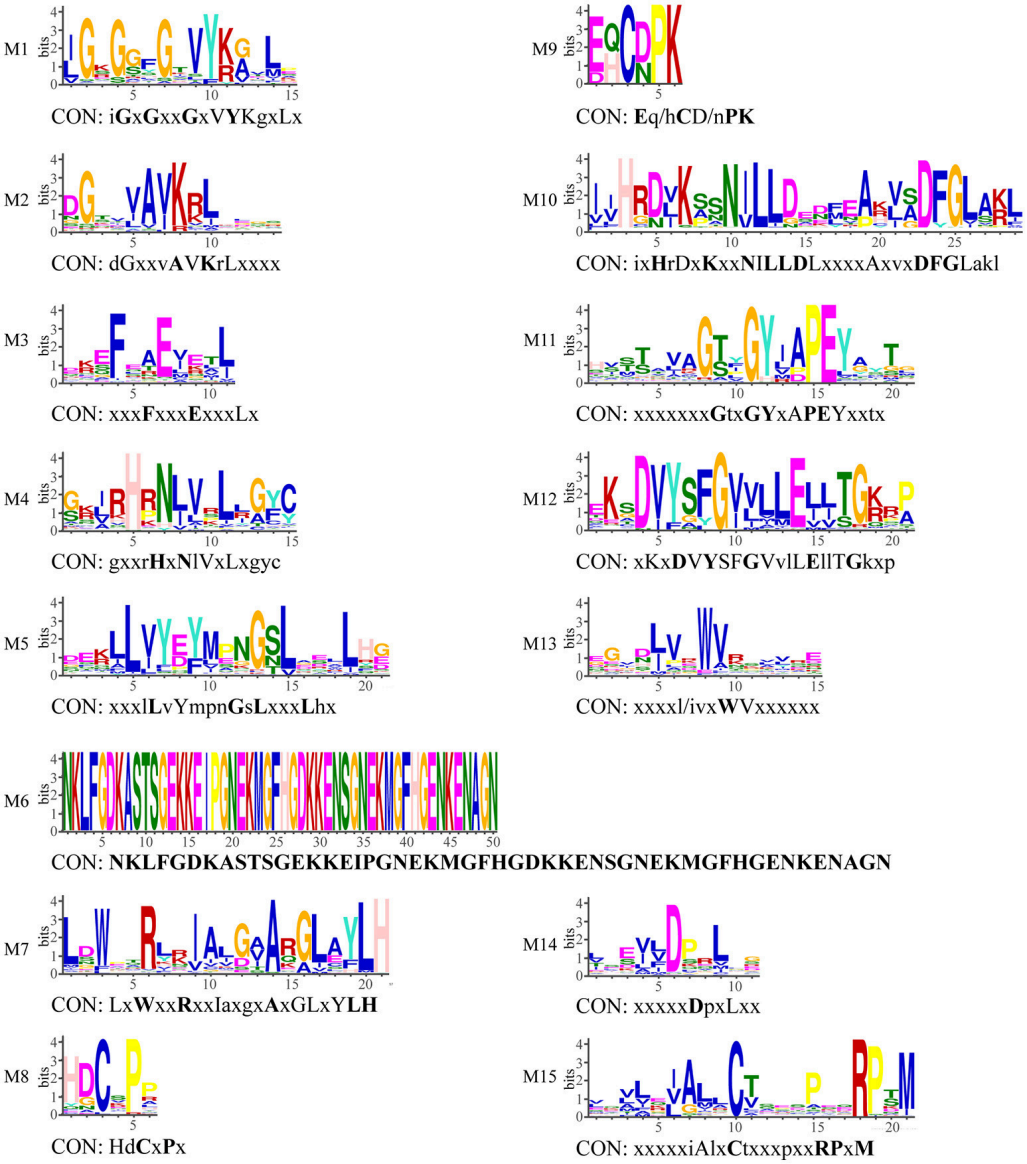
**Conserved motifs in kinase domain of LRR-RLK proteins and their consensus sequences**. CON indicates consensus sequence. If the bits value of amino acid at this position is smaller than 1, it is represent with x; 2 > bits ≥ 1, with lowercase; 3 > bits ≥ 2, with capital letter; bits ≥ 3, with bold capital.

In comparison with the LRR domain, the KD domain is better conserved. To explore evolutionary divergence of the KD domain, we performed motif analyses with the MEME program. MEME analysis identified 15 motifs in the *LRR-RLK* kinase domain (from the N-terminus to the C-terminus): M1 to M15 (Figures 3C, 4). The KD domain can be divided into 12 subdomains: 8 with conserved residues (I, II, III, VIB, VII, VIII, IX, and XI) and 4 without conserved residues (IV, V, VIA, and X) (Hanks et al., 1988; Hanks and Hunter, 1995). Subdomain X is the most poorly conserved subdomain and its function is obscure (Hanks and Hunter, 1995). In the study, 8 conserved subdomains can be found in the MEME motifs according to their position in the kinase domain and their conserved amino acid residues (Figure 4): motifs M1, M2, M3, M10, M11, M12, and M15 correspond to subdomains I, II, III, VIb & VII, VIII, IX, and XI, respectively. These motifs are shared by almost all *LRR-RLK* genes, with the exception of motifs M3 (subdomain III) and M11 (subdomain VIII) (Table 1). Meanwhile, 3 less conserved subdomains can also be found in the MEME motifs only according to their positions: motifs M4, M5, and M7 correspond to subdomains IV, V, and VIa, respectively. These motifs are also shared by all subfamilies and almost all members of each subfamily (Figure 3C, Table 1). M6 also correspond to subdomain V, it was only present in two members of subfamily X^*^, As previous studies, it is difficult to determine the correspondence of less conserved subdomain X. Other four MEME motif (M8, M9, M13 ad M14) do not correspond to the known subdomains. M8 are shared by all members of subfamilies XIII-1 and XIII-2, and most members of subfamilies I and XI^*^. Motif M9 is subfamily-specific, and appeared only in subfamily II. Motif M14 were absent from several members of a few subfamilies and shared by most LRR-RLK proteins. Motif M13 together with M3 (subdomain III), and M11 (subdomain VIII) were absent from a few *LRR-RLK* subfamilies. For example, M3 (subdomain III) was absent from all *LRR-RLK* genes of subfamilies V and VI-2, as well as most of those of subfamilies III, VI-1, and X^*^. Motif M11 (subdomain VIII) was not observed in any *LRR-RLK* genes of subfamilies VI-1 and VI-2, as well as most genes of subfamily III (Figure 3C and Table 1).

Besides, to find all possible domains in LRR-RLK proteins, all proteins identified in this study were analyzed with Pfam (http://pfam.xfam.org/). The results showed that three members in subfamily I and all members in subfamily III each harbored an extracellular malectin-like (ML) domain.

### Genomic structure of *LRR-RLK* genes

We analyzed the gene structures of 92 *LRR-RLK* genes of *A. trichopoda* and mapped the structures to their corresponding genes in the phylogenetic tree (Figure 3) (For Am56.177 and Am56.178, the structrues are unavailable on phytozome). As shown in Figures 3A,D and Table 1, we found that most of the closely related *A. trichopoda* LRR-RLK genes have roughly the same number and position of introns, strongly supporting their close evolutionary relationships. For example, all member of subfamily XII have one intron over their coding sequences, whereas almost all members of subfamily II have ten introns. We also found that *A. trichopoda* and *A. thaliana* LRR-RLK genes belonging to the same subfamily exhibit similar genomic features. For example, 12 of 14 *A. thaliana LRR-RLK* genes of subfamily II have introns with the same numbers and positions over their coding sequences as that of *A. trichopoda*.

### Selection test

To evaluate the selective pressures acting on *LRR-RLK* genes in the selected subfamily, we conducted likelihood ratio tests in three pairs of models. The results are shown in Tables 2, 3. The LRTs for M3 vs. M0 were significant in all cases. The discrete model (M3) with three sites classes revealed a quite homogeneous picture of purifying selection among the sequences of all analyzed subfamilies. The ω values of the three site classes of all analyzed subfamilies were lower than 1 (0–0.1915) (Table 2) with two exceptions. A proportion of sites (30.2%) in subfamily I had a ω ratio of 1.0357 and a proportion of sites (20.8%) in subfamily VIII-1 had a ω ratio of 1.2900. In subfamily I and VIII-1, 22 and 6 sites, respectively, with NEB support >95% were indicated as putative sites under positive selection. However, M2 vs. M1 and M8 vs. M7 were not significant in all cases (Table 3), suggesting that the M1 and M7 models fit the observed data for these subfamilies. The M1 and M7 models do not assume positively selected sites. Nearly neutral model M1 revealed that 93.932–73.751% of the sites in all analyzed subfamilies have a ω ratio lower than 1 (0.0727–0.1643). Therefore, for all subfamilies, purifying selection seems to better explain the data.

**Table 2:** **Codeml site-models parameter values and likelihood scores estimated for each individual selected subfamilies**.

**Subfamily**	**Models and parameter estimates**	**L**
**M0 (ONE RATIO)**		
I	ω_0_ = 0.3743	−11330.92
II	ω_0_ = 0.0920	−11063.62
III	ω_0_ = 0.2292	−40462.98
VIII_1	ω_0_ = 0.2949	−11381.07
XII	ω_0_ = 0.2182	−23228.48
**M1 (NEARLY NEUTRAL)**		
I	(0 < ω_0_ <1, ω_0_ = 0.1112) *p*_0_ = 0.561, (ω_1_ = 1) *p*_1_ = 0.439	−6245.62
II	(0 < ω_0_ <1, ω_0_ = 0.0807) *p*_0_ = 0.738, (ω_1_ = 1) *p*_1_ = 0.262	−10906.03
III	(0 < ω_0_ <1, ω_0_ = 0.1643) *p*_0_ = 0.379, (ω_1_ = 1) *p*_1_ = 0.621	−39806.09
VIII_1	(0 < ω_0_ <1, ω_0_ = 0.0727) *p*_0_ = 0.532, (ω_1_ = 1) *p*_1_ = 0.468	−11239.85
XII	(0 < ω_0_ <1, ω_0_ = 0.1336) *p*_0_ = 0.530, (ω_1_ = 1) *p*_1_ = 0.470	−22876.97
**M2 (POSITIVE SELECTION)**		
I	(0 < ω_0_ <1, ω_0_ = 0.1112) *p*_0_ = 0.561, (ω_1_ = 1) *p*_1_ = 0.265, (ω_2_ >1, ω_2_ = 1) *p*_2_ = 0.173	−6245.62
II	(0 < ω_0_ <1, ω_0_ = 0.0807) *p*_0_ = 0.738, (ω_1_ = 1) *p*_1_ = 0.023, (ω_2_ >1, ω_2_ = 1) *p*_2_ = 0.239	−10906.03
III	(0 < ω_0_ <1, ω_0_ = 0.1643) *p*_0_ = 0.378, (ω_1_ = 1) *p*_1_ = 0.618, (ω_2_ >1, ω_2_ = 78.6307) *p*_2_ = 0.003	−39806.06
VIII_1	(0 < ω_0_ <1, ω_0_ = 0.0727) *p*_0_ = 0.532, (ω_1_ = 1) *p*_1_ = 0.207, (ω_2_ >1, ω_2_ = 1) *p*_2_ = 0.262	−11239.85
XII	(0 < ω_0_ <1, ω_0_ = 0.1336) *p*_0_ = 0.530, (ω_1_ = 1) *p*_1_ = 0.342, (ω_2_ >1, ω_2_ = 1) *p*_2_ = 0.129	−22876.97
**M3 (DISCRETE)**		
I	(ω_0_ = 0) *p*_0_ = 0.199, (ω_1_ = 0.2468) *p*_1_ = 0.499, (ω_2_ = 1.0357) *p*_2_ = 0.302	−6236.24
II	(ω_0_ = 0) *p*_0_ = 0.233, (ω_1_ = 0.0582) *p*_1_ = 0.406, (ω_2_ = 0.2836) *p*_2_ = 0.362	−10795.06
III	(ω_0_ = 0.0437) *p*_0_ = 0.194, (ω_1_ = 0.2050) *p*_1_ = 0.282, (ω_2_ = 0.4979) *p*_2_ = 0.524	−39489.33
VIII_1	(ω_0_ = 0.0157) *p*_0_ = 0.288, (ω_1_ = 0.2942) *p*_1_ = 0.504, (ω_2_ = 1.2900) *p*_2_ = 0.208	−11218.68
XII	(ω_0_ = 0.0140) *p*_0_ = 0.189, (ω_1_ = 0.1749) *p*_1_ = 0.413, (ω_2_ = 0.5757) *p*_2_ = 0.398	−22754.11
**M7 (BETA)**		
I	*P* = 0.465, *q* = 0.682	−6238.20
II	*P* = 0.49967, *q* = 3.125	−10794.58
III	*P* = 0.932, *q* = 1.660	−39479.63
VIII_1	*P* = 0.411, *q* = 0.673	−11221.78
XII	*P* = 0.697, *q* = 1.518	−22753.97
**M8 (BETA AND W)**		
I	*p* = 0.584, *q* = 1.410, *p*_0_ = 0.833, (ω_1_ = 1.1338), *p*_1_ = 0.167	−6237.41
II	*p* = 0.506, *q* = 3.249, *p*_0_ = 0.995, (ω_1_ = 2.1221), *p*_1_ = 0.005	−10794.53
III	*p* = 0.932, *q* = 1.661, *p*_0_ = 0.99999, (ω_1_ = 1.0000), *p*_1_ = 0.00001	−39479.63
VIII_1	*p* = 0.460, *q* = 0.940, *p*_0_ = 0.941, (ω_1_ = 2.0703), *p*_1_ = 0.059	−11219.10
XII	*p* = 0.690, *q* = 1.611, *p*_0_ = 0.977, (ω_1_ = 3.2665), *p*_1_ = 0.023	

**Table 3:** **Likelihood ratio test of positive selection in LRR-RLK subfamily proteins**.

**Subfamily**	**2L/M3 vs. MO**	**2L/M2a vs. M1a**	**2L/M8 vs. M7**
I	306.86551^*^	0	5.479124
II	537.108832^*^	0	0.101476
III	1947.3058^*^	0.69886	0.00078
VIII_1	324.776964^*^	0	5.357916
XII	948.721848^*^	0	0.353648

* *indicates significant at p < 0.001 level*.

### Differential expression analysis of *A. trichopoda* LRR-RLK genes

**Figure 5:**
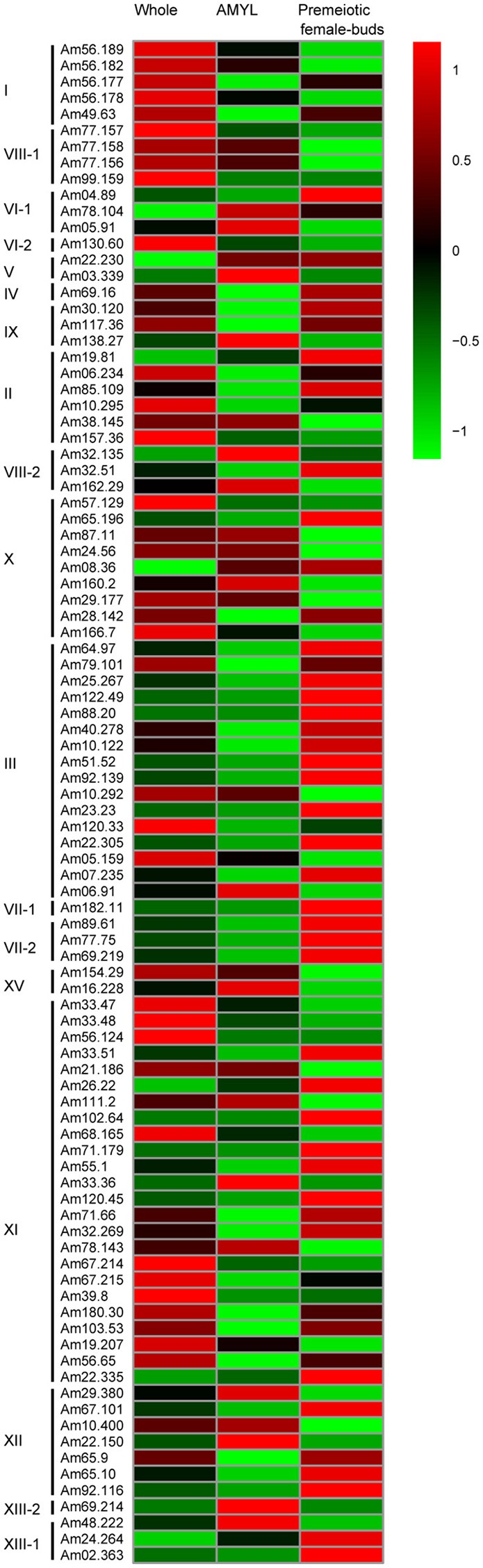
**Transcript abundance of 94 ***A. trichopoda LRR-RLK*** genes**. The genes were grouped according to subfamily and the color scale represents the expression values. AMYL indicates apical meristem and young leaves.

To understand the putative functions of *LRR-RLK* genes in *A. trichopoda*, the expression profiles of these genes were examined by using the RNA-seq data from three tissues: whole plant, apical meristem and young leaves (AMYL), and pre-meiotic female flower buds. As shown in the heat map (Figure 5), all *LRR-RLK* genes in subfamilies I, II, VI-2, and VIII-1showed high expression levels in whole plant, while all *LRR-RLK* genes in subfamilies V, VI-1, VI-1, VII-2, VIII-2, XIII-1, and XIII-2 showed low expression levels in whole plant. Genes from other subfamilies, such as III, X^*^ and XI^*^, had very different expression levels in whole plant: some had high expression levels, some had moderate expression levels and some showed very low expression levels. In apical meristem and young leaves, we found 28 genes were highly expressed. These genes included all genes from subfamilies V, XIII-2, and XV and some genes from subfamilies II, III, VI-1, VIII-1, VIII-2, IX, X^*^, XI^*^, XII, and XIII-1. In pre-meiotic female flower buds, 45 genes showed high expression levels, six showed moderate expression levels and others showed low expression levels. The highly expressed 45 genes included all genes from subfamilies IV, VII-1, and VII-2, majority of genes from subfamilies III, V, IX, XI^*^, XII, and XIII-1, and some genes from subfamilies II, VI-1, VIII-2, and X^*^.

## Discussion

*LRR-RLK* genes have been identified in some eudicots through analysis of genome sequences. For example, in genomes of *A. thaliana, B. rapa, Citrus clementina* and *Citrus sinensis, S. lycopersicum* and *P. trichocarpa*, 213, 303, 300, 297, 234, and 379 *LRR-RLK* genes, respectively, have been identified (Shiu and Bleecker, 2001; Zan et al., 2013; Rameneni et al., 2015; Wei et al., 2015; Magalhães et al., 2016). In *G. max* genome, as many as 467 genes were identified (Zhou et al., 2016). In monocot *O. sativa* genome, previous studies also identified 309 LRR-RLK genes (Sun and Wang, 2011). In another monocot *T. aestivum* genome, 531 LRR-RLK genes were identified, which showed the largest copy number of *LRR-RLK* gene family as far as we know (Shumayla et al., 2016). A recent study showed there are 7, 554 *LRR-RLK* genes in 31 fully sequenced flowering plant genomes, with mean 243 *LRR-RLK* genes in each angiosperm genome. This study estimated that the copy number of ancestral genes present in the last common ancestor of angiosperm (exactly is common ancestor of eudicots and monocots since their analyses only included data from eudicot and monocots) is 150. However, in the present study, we only identified 94 *LRR-RLK* genes in *A. trichopoda*. The number of *LRR-RLK* genes in the last common ancestor of eudicots and monocots is 1.60 times (150/94) that of *A. trichopoda*, and the number of *LRR-RLK* genes in eudicots and monocots is roughly 2–6 times that of *A. trichopoda*,. The difference in the numbers of *LRR-RLK* genes between *A. trichopoda* and the ancestor of eudicots/monocots, and between *A. trichopoda* and eudicots and monocots, suggests a relatively greater degree of lineage-specific expansion of this gene family in the lineages leading to the ancestor of eudicots/monocots and to eudicots and monocots. Indeed, Fischer et al. (2016) demonstrated that the expansion rates of *LRR-RLK* genes are very dynamic in angiosperm (eudicots + monocots) and *LRR-RLK* genes showed some degree of expansion in most species. When we compared the copy number of each subfamily between *A. trichopoda* and *A. thaliana*, in consistent with previous studies (Fischer et al., 2016), we found the expansion rates of different subfamilies varied and subfamilies I, VI-2, VIII-2, and XIII-2 showed the largest expansion rates to the lineage to Brassicaceae (Table 1). There are at least four major mechanisms that produce duplicate genes: tandem gene duplication, whole genome duplication (WGD), segmental duplication, and transpositional duplication (Freeling, 2009). Previous studies demonstrated that tandem duplication and WGD played a major role in expansion of the *LRR-RLK* gene family in some eudicot, such as in *A. thaliana, P. trichocarpa, G. max*, and monocot *O. sativa* (Shiu and Bleecker, 2001, 2003; Sun and Wang, 2011; Zan et al., 2013; Zhou et al., 2016). Genes in the same cluster showed more similarity to each other, suggesting that tandem duplication events should be responsible for the origin of the genes in each cluster. In *P. trichocarpa, G. Max* and *O. sativa*, 82, 20.3, and 45% of genes, respectively were derived from tandem duplication after whole-genome duplication (Sun and Wang, 2011; Zan et al., 2013; Zhou et al., 2016). However, after examination of the locations of *LRR-RLK* genes on the chromosomes of *A. trichopoda*, we determined that only 12.8% of *LRR-RLK* genes in *A. trichopoda* (Figure 1) may have been derived from tandem duplication. Thus, unlike the cases in some eudicots and monocot rice, tandem duplication seemed to play a minor role for the formation of the *LRR-RLK* gene family in *A. trichopoda*. WGD or polyploidy is prominent in the evolutionary history of angiosperms (Soltis et al., 2009). For example, there have probably been at least two or three rounds of paleo-polyploidization in the evolution of the *O. sativa* and *A. thaliana* lineages since their splits (Blanc et al., 2003; Bowers et al., 2003; Paterson et al., 2004). In *P. trichocarpa* and *G. Max*, 20 and 73.3% of *LRR-RLK* genes were located in WGD regions (Zan et al., 2013; Zhou et al., 2016). In rice, at least 15 pairs of *LRR-RLK* genes (9.7%) were located on the retention regions after genome duplication (Sun and Wang, 2011). However, with the exception of the common ancient WGD that occurred shortly before the diversification of all living angiosperms, the *A. trichopoda* genome shows no evidence of lineage-specific WGD (Albert et al., 2013). According the study of *A. trichopoda* genome sequences, 47 intra-*Amborella* syntenic block were identified containing 466 gene pairs (Table S10 showed the syntenic gene pairs in Albert et al., 2013). All the syntenic, duplicated blocks correspond to the common ancient WGD that occurred shortly before the diversification of all living angiosperms, and none correspond to segmental duplication. Hence, the three pairs of genes we found in these syntenic blocks (Am04.89 and Am78.104; Am68.165 and Am71.179; Am24.264 and Am48.222) were the result of WGD but not the result of segmental duplication. Therefore, tandem duplication and WGD have made limited contributions to the expansion of *LRR-RLK* genes in *A. trichopoda* while segmental duplication did not contribute to the expansion of *LRR-RLK* genes in *A. trichopoda*. The rarity of lineage-specific WGD/segmental duplication and tandem duplication events in *A. trichopoda* may explain why *A. trichopoda* has few LRR-RLK genes in comparison with eudicot and monocot plants. Then, what is the main contributer to *LRR-RLK* duplication in *A. trichopoda*? Single gene transposition duplications exist in plants, but they are incompletely understood (Freeling, 2009). Considering that most genes position in different scaffolds, transposition may be common in the evolution of *A. trichopoda LRR-RLK* genes. It would be interesting to study this in more detail in the future.

After duplication, duplicated genes often accumulate mutations leading to functional divergence. Although few functional studies of *LRR-RLK* genes in *A. trichopoda* have been performed, diversification of the *LRR-RLK* genes of *A. trichopoda* can be deduced from phylogenetic analysis, protein structures, gene structure and expression profile analysis. Phylogenetic analyses have classified the diversity of *LRR-RLK* genes of *A. trichopoda* into 18 distinct subfamilies (Figure 2A), which largely correspond to subfamilies generated from phylogenetic analysis of *A. thaliana* (Shiu and Bleecker, 2001). As shown in the phylogenetic tree (Figure 2B), *A. trichopoda LRR-RLK* sequences occurred in almost every major clade that was defined as a subfamily in *A. thaliana* (Figure 2). This result suggested that almost all *A. thaliana LRR-RLK* subfamilies existed in the most recent common ancestor of extent angiosperms, which also suggested the duplication and diversification of *LRR-RLK* genes occurred before the origin of extant angiosperms. In phylogenetic trees, sequences in the same clade (or subfamily) usually have relatively similar functions. According to the functional studies, we know different subfamily members of *LRR-RLK* gene in *A. thaliana* usually have different functions. For example, *A. thaliana FRK* gene in subfamily I is involved in defense signaling (Asai et al., 2002); *SEEK1-2, BAK1* and *BKK1* in subfamily II are involved in somatic embryogenesis and brassinosteroid signaling (He et al., 2007); *PXC1* gene in subfamily III is involved in secondary cell wall formation in xylem fiber (Wang et al., 2013) and *SUB*/*SCM* gene in subfamily V regulated organ development (Chevalier et al., 2005). Hence, members of different subfamilies of *LRR-RLK* genes of *A. trichopoda* may have different functions. The phylogenetic analysis indicated functional divergence of *LRR-RLK* gene subfamilies in *A. trichopoda*.

The function of a protein is linked to its amino acid sequence. The protein structure characteristic of LRR-RLK proteins have been demonstrated in many studies (Shiu and Bleecker, 2001). They compose of three functional domains: the extracellular LRR domain, a transmembrane domain, and an intracellular kinase domain (Shiu and Bleecker, 2001). In this study, we focused on the number of LRR repeats in the LRR domain and motifs in the KD domain. Previous studies in eudicots and monocots reported that members within the same LRR-RLK subfamily tend to have a similar number of LRR repeats, whereas members of different subfamilies exhibit a different number of LRR repeats (Shiu and Bleecker, 2001; Sun and Wang, 2011; Zan et al., 2013; Rameneni et al., 2015; Wei et al., 2015; Magalhães et al., 2016; Shumayla et al., 2016). The protein structure analyses of LRR-RLK proteins in *A. trichopoda* reinforced the conclusion that the same subfamily members tend to have broadly the same number of LRR repeats in the LRR domain (Figures 3A,B and Table 1), while members of different subfamilies have different numbers of LRR repeats in the LRR domain. For example, most members of subfamily I have 3 LRR repeats, all members of subfamily II have 3 or 4 LRR repeats, and most members of subfamily VIII-1 have 11 LRR repeats. In addition, members of subfamilies X^*^ and XI^*^ tended to have many more LRR repeats. It has demonstrated that the number of LRR repeats have a significant impact on LRR-RLK proteins contained them. Usually, RLK proteins with few LRRs are more likely to be co-receptors or cofactors (Somssich et al., 2016). For example, the perception of brassinosteroids (BRs) and flg 22 by their LRR-kinase receptor BRI1 and FLS2 often results in the recruitment of a co-receptor, such as BAK1 (Li et al., 2002; Sun et al., 2013a,b); The receptors BRI1 in LRR-RLK subfamily X^*^ contains 25 LRRs and FLS2 in LRR-RLK subfamily XII contained 28 LRRs; however, the co-receptor BAK1 (SERK3) in LRR-RLK subfamily II contain a shorter LRR, 4 LRRs. Moreover, LRR domains interaction with different substrates (including proteins, nucleic acids, lipids, and small molecule hormones) showed different LRR numbers and arrangements (Helft et al., 2011). Hence, the divergence of LRRs among different subfamilies appears to reflect their divergence with respect to ligand perception.

When the LRR domain binds a ligand, the KD is activated to trigger activation of downstream substrates (Gou et al., 2010). The KDs contain 12 conserved subdomains (Hanks et al., 1988; Hanks and Hunter, 1995). These subdomains often contain conserved residues (except for subdomains IV, V, VIa, and X), which play important roles in enzyme function (Hanks et al., 1988; Hanks and Hunter, 1995; Krupa et al., 2004). In the present study, we identified 15 motifs through MEME motif analysis (Figure 4). Eight motifs (M1, M2, M4, M5, M7, M10, M12, and M15) are shared by essentially all LRR-RLK proteins identified in *A. trichopoda* (Figure 3C and Table 1). M1, M2, M10, M12, and M15 correspond to subdomains with conserved residues (I, II, VIb & VII, IX, and XI), whereas M4, M5, and M7 correspond to less conserved subdomains (IV, V, and VIa). These common motifs indicate functional similarities in kinase activity. The MEME analysis also showed that some motifs are present only in some subfamilies, suggesting functional divergence. For example, subdomain III (motif M3) of the KD contains a nearly invariant Glu residue that is required for kinase activity (Hanks et al., 1988; Hanks and Hunter, 1995). The absence of M3 from all members of subfamilies V and VI-2, as well as most members of subfamily III, suggests significant functional divergence in the kinase activity of these subfamily members from those containing M3. Indeed, biochemical assays of the SUB (one member of subfamily V) kinase domain, suggested that it lacks enzymatic phosphotransfer activity (Chevalier et al., 2005). In addition, we also identified one subfamily-specific motif, M9, which appeared only in subfamily II and may contribute to the functional divergence of this subfamily.

It was noted that except the LRR-TM-Kinase motifs, we also identified an malectin-like (ML) domain before a short stretch of LRR repeats in three members of subfamily I and all members of subfamily VIII-1. According to the previous study, the malectin-like domain is likely involved in carbohydrate binding (Schallus et al., 2008). They are found in RLK from plants and in protein described as glycoside hydrolases. One member with malectin-like domain from *LRR-RLK* subfamily I of *A. thaliana*, ISO1, confers susceptibility to a downy mildew pathogen in *A. thaliana* (Hok et al., 2011). Although the function of malectin-like domains of RLK is not well understood (Lindner et al., 2012), the extra malectin-like domain in these two subfamilies may suggested a significant functional divergence of them from other subfamilies.

According to the protein structure and motif analyses, we concluded that the protein sequences of duplicated *LRR-RLK* gene diversified during evolution. Therefore, we assessed whether relaxation of purifying selection or positive selection was the major cause of sequence diversification of the duplicated genes. Selection test showed that neutral models M1 and discrete M7 fit the data significantly better than the other tested models. Nearly neutral model M1 revealed that 73.751–93.932% of the sites in all analyzed subfamilies had a ω ratio less than 1 (0.0727–0.1643). Therefore, for all of the analyzed subfamilies, most sites were under negative or relaxed purifying selection. This finding was largely consistent with the results of selection testing of the *LRR-RLK* subfamilies of *O. sativa* (Sun and Wang, 2011).

Like the protein structure of the *LRR-RLK* genes, their gene structure showed significant diversification between the subfamilies of *LRR-RLK* proteins in *A. trichopoda*. We found that most of the closely related *A. trichopoda LRR-RLK* genes have roughly the same number and location of introns, which strongly supports their close evolutionary relationship. However, different subfamily members show different intron/exon structures. For example, all member of subfamily XII have one intron over their coding sequences, while most members of subfamily II have ten introns. Introns play important roles in various cellular and developmental processes via alternate splicing or regulation of gene expression (Roy and Gilbert, 2006). The presence of multiple introns of *LRR-RLK* gene *ERECTA* has been demonstrated to be essential for its expression in *A. thaliana* (Karve et al., 2011). The unique structures of each subfamily provide additional evidence that supports functional divergence between LRR-RLK subfamilies.

Tissue-specific transcript abundance is suggestive of a genes biological function. Gene expression patterns of LRR-RLK genes also showed significant diversification of genes from different subfamilies. For example, all LRR-RLK genes in subfamilies I, II, VI-2, and VIII-1 showed high expression levels; conversely, a low expression level was observed for all LRR-RLK genes in subfamilies V, VI-1, VI-1, VII-2, VIII-2, XIII-1, and XIII-2 in whole plant (Figure 5). All genes from subfamilies V, XIII-2, and XV were highly expressed in apical meristem and young leaves, and all genes from subfamilies IV, VII-1, and VII-2 and most genes from subfamilies III, V, IX, XI^*^, and XII were highly expressed in pre-meiotic female flower buds. Besides, genes even from the same subfamily also showed different expression patterns. Some genes from subfamilies III, X^*^, and XI^*^ showed high expression levels in whole plant, some showed moderate expression levels and some showed very low expression levels in that tissue (Figure 5). Some genes from subfamilies II, III, VI-1, VIII-1, VIII-2, IX, X^*^, XI^*^, XII, and XIII-1 showed high expression levels in apical meristem and young leaves, and some genes from these subfamilies showed low expression levels in that tissue. In pre-meiotic female flower buds, one-third of genes from subfamilies II, VI-1, VIII-2, and X^*^ showed high expression levels, whereas two-third of genes from these subfamilies showed low expression levels. Hence, the results suggested *LRR-RLK* genes from different subfamilies and even genes from the same subfamilies exhibited expressional divergence. Similar results have also been obtained from the expression analyses of *LRR-RLK* genes from other plants (Zan et al., 2013; Rameneni et al., 2015; Wei et al., 2015; Shumayla et al., 2016; Zhou et al., 2016).

Taken together, our phylogenetic analysis, protein structure and motif analysis, gene structure analysis and expression profiling analysis suggest divergence of the *LRR-RLK* subfamilies of *A. trichopoda*. The results of this study reveal the complexity of the LRR-RLK gene family in angiosperm and provide a framework for further functional investigation of *A. trichopoda LRR-RLK* genes.

## Author contributions

P-LL and LX designed the study. P-LL, P-WL, JM, HL, SG, PS, and JG, collected and analyzed the data. P-LL, LX, and P-WL drafted the manuscript.

## Funding

This work was supported by the Fundamental Research Funds for the Central Universities (BLX2013022) and the National Natural Science Foundation of China (31500178).

### Conflict of interest statement

The authors declare that the research was conducted in the absence of any commercial or financial relationships that could be construed as a potential conflict of interest.

## Supplementary material

Supplemental Figure 1 **Phylogeny of LRR-RLK genes in ***Amborella trichopoda*****. This phylogenetic tree based on kinase domain sequence was constructed by the Maximum Likelihood method.

Supplemental Figure 2 **Phylogeny of LRR-RLK genes in ***Amborella trichopoda*** and ***Arabidopsis thaliana*****. This phylogenetic tree based on kinase domain sequence was constructed by the Maximum Likelihood method.

Supplemental Table 1 **The full name of 94 LRR-RLK proteins identified in the ***Amborella trichopoda*** genome**.

## References

[B1] Agusti J. Lichtenberger R. Schwarz M. Nehlin L. Greb T. (2011). Characterization of transcriptome remodeling during cambium formation identifies *MOL1* and *RUL1* as opposing regulators of secondary growth. PLoS Genet. 7:e1001312. 10.1371/journal.pgen.1001312 21379334 PMC3040665

[B2] Albert V. A. Barbazuk W. B. dePamphilis C. W. Der J. P. Leebens-Mack J. Ma H.. (2013). The *Amborella* genome and the evolution of flowering plants. Science 342:1467. 10.1126/science.1241089 24357323

[B3] Albrecht C. Russinova E. Kemmerling B. Kwaaitaal M. de Vries S. C. (2008). Arabidopsis SOMATIC EMBRYOGENESIS RECEPTOR KINASE proteins serve brassinosteroid-dependent and -independent signaling pathways. Plant Physiol. 148, 611–619. 10.1104/pp.108.123216 18667726 PMC2528080

[B4] Asai T. Tena G. Plotnikova J. Willmann M. R. Chiu W. L. Gomez-Gomez L.. (2002). MAP kinase signalling cascade in *Arabidopsis* innate immunity. Nature 415, 977–983. 10.1038/415977a 11875555

[B5] Bailey T. L. Boden M. Buske F. A. Frith M. Grant C. E. Clementi L.. (2009). MEME SUITE: tools for motif discovery and searching. Nucleic Acids Res. 37, W202–W208. 10.1093/nar/gkp335 19458158 PMC2703892

[B6] Blanc G. Hokamp K. Wolfe K. H. (2003). A recent polyploidy superimposed on older large-scale duplications in the *Arabidopsis* genome. Genome Res. 13, 137–144. 10.1101/gr.751803 12566392 PMC420368

[B7] Bojar D. Martinez J. Santiago J. Rybin V. Bayliss R. Hothorn M. (2014). Crystal structures of the phosphorylated BRI1 kinase domain and implications for brassinosteroid signal initiation. Plant J. 78, 31–43. 10.1111/tpj.12445 24461462 PMC4260089

[B8] Bolger A. M. Lohse M. Usadel B. (2014). Trimmomatic: a flexible trimmer for Illumina sequence data. Bioinformatics 30, 2114–2120. 10.1093/bioinformatics/btu170 24695404 PMC4103590

[B9] Bowers J. E. Chapman B. A. Rong J. K. Paterson A. H. (2003). Unravelling angiosperm genome evolution by phylogenetic analysis of chromosomal duplication events. Nature 422, 433–438. 10.1038/nature01521 12660784

[B10] Chang F. Gu Y. Ma H. Yang Z. (2013). AtPRK2 promotes ROP1 activation via RopGEFs in the control of polarized pollen tube growth. Mol. Plant 6, 1187–1201. 10.1093/mp/sss103 23024212 PMC3888354

[B11] Chevalier D. Batoux M. Fulton L. Pfister K. Yadav R. K. Schellenberg M.. (2005). *STRUBBELIG* defines a receptor kinase-mediated signaling pathway regulating organ development in *Arabidopsis*. Proc. Natl. Acad. Sci. U.S.A. 102, 9074–9079. 10.1073/pnas.0503526102 15951420 PMC1157047

[B12] Clark S. E. Williams R. W. Meyerowitz E. M. (1997). The *CLAVATA1* gene encodes a putative receptor kinase that controls shoot and floral meristem size in Arabidopsis. Cell 89, 575–585. 10.1016/s0092-8674(00)80239-1 9160749

[B13] Edgar R. C. (2004). MUSCLE: multiple sequence alignment with high accuracy and high throughput. Nucleic Acids Res. 32, 1792–1797. 10.1093/nar/gkh340 15034147 PMC390337

[B14] Eyüeboglu B. Pfister K. Haberer G. Chevalier D. Fuchs A. Mayer K. F. X.. (2007). Molecular characterisation of the *STRUBBELIG-RECEPTOR* family of genes encoding putative leucine-rich repeat receptor-like kinases in *Arabidopsis thaliana*. BMC Plant Biol. 7:16. 10.1186/1471-2229-7-16 17397538 PMC1855328

[B15] Fischer I. Diévart A. Droc G. Dufayard J. F. Chantret N. (2016). Evolutionary dynamics of the leucine-rich repeat receptor-like kinase (LRR-RLK) subfamily in angiosperms. Plant Physiol. 170, 1595–1610. 10.1104/pp.15.01470 26773008 PMC4775120

[B16] Fontes E. P. B. Santos A. A. Luz D. F. Waclawovsky A. J. Chory J. (2004). The geminivirus nuclear shuttle protein is a virulence factor that suppresses transmembrane receptor kinase activity. Genes Dev. 18, 2545–2556. 10.1101/gad.1245904 15489295 PMC529541

[B17] Freeling M. (2009). Bias in plant gene content following different sorts of duplication: tandem, whole-genome, segmental, or by transposition. Annu. Rev. Plant Biol. 60, 433–453. 10.1146/annurev.arplant.043008.092122 19575588

[B18] Gómez-Gómez L. Boller T. (2000). FLS2: an LRR receptor-like kinase involved in the perception of the bacterial elicitor flagellin in *Arabidopsis*. Mol. Cell 5, 1003–1011. 10.1016/s1097-2765(00)80265-8 10911994

[B19] Goodstein D. M. Shu S. Q. Howson R. Neupane R. Hayes R. D. Fazo J.. (2012). Phytozome: a comparative platform for green plant genomics. Nucleic Acids Res. 40, D1178–D1186. 10.1093/nar/gkr944 22110026 PMC3245001

[B20] Gou X. He K. Yang H. Yuan T. Lin H. Clouse S. D.. (2010). Genome-wide cloning and sequence analysis of leucine-rich repeat receptor-like protein kinase genes in *Arabidopsis thaliana*. BMC Genomics 11:19. 10.1186/1471-2164-11-19 20064227 PMC2817689

[B21] Hanks S. K. Hunter T. (1995). Protein kinases 6. The eukaryotic protein kinase superfamily: kinase (catalytic) domain structure and classification. FASEB J. 9, 576–596. 7768349

[B22] Hanks S. K. Quinn A. M. Hunter T. (1988). The protein kinase family: conserved features and deduced phylogeny of the catalytic domains. Science 241, 42–52. 10.1126/science.3291115 3291115

[B23] He K. Gou X. P. Yuan T. Lin H. H. Asami T. Yoshida S.. (2007). BAK1 and BKK1 regulate Brassinosteroid-dependent growth and Brassinosteroid Independent cell-death pathways. Curr. Biol. 17, 1109–1115. 10.1016/j.cub.2007.05.036 17600708

[B24] Helft L. Reddy V. Chen X. Koller T. Federici L. Fernández-Recio J.. (2011). LRR conservation mapping to predict functional sites within protein leucine-rich repeat domains. PLoS ONE 6:e21614. 10.1371/journal.pone.0021614 21789174 PMC3138743

[B25] Hok S. Danchin E. G. J. Allasia V. Panabiéres F. Attard A. Keller H. (2011). An Arabidopsis (malectin-like) leucine-rich repeat receptor-like kinase contributes to downy mildew disease. Plant Cell and Environ. 34, 1944–1957. 10.1111/j.1365-3040.2011.02390.x 21711359

[B26] Hu B. Jin J. Guo A.-Y. Zhang H. Luo J. Gao G. (2015). GSDS 2.0: an upgraded gene feature visualization server. Bioinformatics 31, 1296–1297. 10.1093/bioinformatics/btu817 25504850 PMC4393523

[B27] Karve R. Liu W. Willet S. G. Torii K. U. Shpak E. D. (2011). The presence of multiple introns is essential for *ERECTA* expression in *Arabidopsis*. RNA 17, 1907–1921. 10.1261/rna.2825811 21880780 PMC3185922

[B28] Kolde R. (2012). Pheatmap: Pretty Heatmaps. R package version.

[B29] Krogh A. Larsson B. von Heijne G. Sonnhammer E. L. (2001). Predicting transmembrane protein topology with a hidden Markov model: application to complete genomes. J. Mol. Biol. 305, 567–580. 10.1006/jmbi.2000.4315 11152613

[B30] Krupa A. Preethl G. Srinivasan N. (2004). Structural modes of stabilization of permissive phosphorylation sites in protein kinases: distinct strategies in Ser/Thr and Tyr kinases. J. Mol. Biol. 339, 1025–1039. 10.1016/j.jmb.2004.04.043 15178245

[B31] Lehti-Shiu M. D. Shiu S.-H. (2012). Diversity, classification and function of the plant protein kinase superfamily. Philos. Trans. R. Soc. B 367, 2619–2639. 10.1098/rstb.2012.0003 22889912 PMC3415837

[B32] Lehti-Shiu M. D. Zou C. Hanada K. Shiu S.-H. (2009). Evolutionary history and stress regulation of plant receptor-like kinase/Pelle genes. Plant Physiol. 150, 12–26. 10.1104/pp.108.134353 19321712 PMC2675737

[B33] Li H. (2013). Aligning sequence reads, clone sequences and assembly contigs with BWA-MEM. arXiv:13033997.

[B34] Li J. Chory J. (1997). A putative leucine-rich repeat receptor kinase involved in brassinosteroid signal transduction. Cell 90, 929–938. 10.1016/s0092-8674(00)80357-8 9298904

[B35] Li J. Wen J. Lease K. A. Doke J. T. Tax F. E. Walker J. C. (2002). BAK1, an Arabidopsis LRR receptor-like protein kinase, interacts with BRI1 and modulates brassinosteroid signaling. Cell 110, 213–222. 10.1016/S0092-8674(02)00812-7 12150929

[B36] Lindner H. Müeller L. M. Boisson-Dernier A. Grossniklaus U. (2012). CrRLK1L receptor-like kinases: not just another brick in the wall. Curr. Opin. Plant Biol. 15, 659–669. 10.1016/j.pbi.2012.07.003 22884521

[B37] Llorente F. Alonso-Blanco C. Sánchez-Rodriguez C. Jorda L. Molina A. (2005). ERECTA receptor-like kinase and heterotrimeric G protein from *Arabidopsis* are required for resistance to the necrotrophic fungus Plectosphaerella cucumerina. Plant J. 43, 165–180. 10.1111/j.1365-313X.2005.02440.x 15998304

[B38] Magalhães D. M. Scholte L. L. S. Silva N. V. Oliveira G. C. Zipfel C. Takita M. A.. (2016). *LRR-RLK* family from two *Citrus* species: genome-wide identification and evolutionary aspects. BMC Genomics 17:623. 10.1186/s12864-016-2930-9 27515968 PMC4982124

[B39] Marchler-Bauer A. Lu S. N. Anderson J. B. Chitsaz F. Derbyshire M. K. DeWeese-Scott C.. (2011). CDD: a conserved domain database for the functional annotation of proteins. Nucleic Acids Res. 39, D225–D229. 10.1093/nar/gkq1189 21109532 PMC3013737

[B40] Muschietti J. Eyal Y. McCormick S. (1998). Pollen tube localization implies a role in pollen-pistil interactions for the tomato receptor-like protein kinases lePRK1 and lePRK2. Plant Cell 10, 319–330. 9501107 10.1105/tpc.10.3.319 PMC143994

[B41] Nodine M. D. Bryan A. C. Racolta A. Jerosky K. V. Tax F. E. (2011). A few standing for many: embryo receptor-like kinases. Trends Plant Sci. 16, 211–217. 10.1016/j.tplants.2011.01.005 21349757

[B42] Nodine M. D. Yadegari F. Tax F. E. (2007). RPK1 and TOAD2 are two receptor-like kinases redundantly required for *Arabidopsis* embryonic pattern formation. Dev. Cell 12, 943–956. 10.1016/j.devcel.2007.04.003 17543866

[B43] Osakabe Y. Maruyama K. Seki M. Satou M. Shinozaki K. Yamaguchi-Shinozaki K. (2005). Leucine-rich repeat receptor-like kinase1 is a key membrane-bound regulator of abscisic acid early signaling in *Arabidopsis*. Plant Cell 17, 1105–1119. 10.1105/tpc.104.027474 15772289 PMC1087989

[B44] Paterson A. H. Bowers J. E. Chapman B. A. (2004). Ancient polyploidization predating divergence of the cereals, and its consequences for comparative genomics. Proc. Natl. Acad. Sci. U.S.A. 101, 9903–9908. 10.1073/pnas.0307901101 15161969 PMC470771

[B45] Rameneni J. J. Lee Y. Dhandapani V. Yu X. Choi S. R. Oh M.-H.. (2015). Genomic and post-translational modification analysis of leucine-rich-repeat receptor-like kinases in *Brassica rapa*. PLoS ONE 10:e0142255. 10.1371/journal.pone.0142255 26588465 PMC4654520

[B46] Roy S. W. Gilbert W. (2006). The evolution of spliceosomal introns: patterns, puzzles and progress. Nat. Rev. Genet. 7, 211–221. 10.1038/nrg1807 16485020

[B47] Schallus T. Jaeckh C. Fehér K. Palma A. S. Liu Y. Simpson J. C.. (2008). Malectin: a novel carbohydrate-binding protein of the endoplasmic reticulum and a candidate player in the early steps of protein N-glycosylation. Mol. Biol. Cell 19, 3404–3414. 10.1091/mbc.E08-04-0354 18524852 PMC2488313

[B48] Shiu S. H. Bleecker A. B. (2001). Receptor-like kinases from *Arabidopsis* form a monophyletic gene family related to animal receptor kinases. Proc. Natl. Acad. Sci. U.S.A. 98, 10763–10768. 10.1073/pnas.181141598 11526204 PMC58549

[B49] Shiu S. H. Bleecker A. B. (2003). Expansion of the receptor-like kinase/Pelle gene family and receptor-like proteins in *Arabidopsis*. Plant Physiol. 132, 530–543. 10.1104/pp.103.021964 12805585 PMC166995

[B50] Shumayla S. Kumar R. Mendu V. Singh K. Upadhyay S.K. (2016). Genomic dissection and expression profiling revealed functional divergence in *Triticum aestivum* leucine rich repeat receptor like kinases (TaLRRKs). Front. Plant Sci. 7:1374. 10.3389/fpls.2016.01374 27713749 PMC5031697

[B51] Soltis D. E. Albert V. A. Leebens-Mack J. Bell C. D. Paterson A. H. Zheng C. F.. (2009). Polyploidy and angiosperm diversification. Am. J. Bot. 96, 336–348. 10.3732/ajb.0800079 21628192

[B52] Somssich M. Je B. I. Simon R. Jackson D. (2016). CLAVATA-WUSCHEL signaling in the shoot meristem. Development 143, 3238–3248. 10.1242/dev.133645 27624829

[B53] Stamatakis A. Hoover P. Rougemont J. (2008). A rapid bootstrap algorithm for the RAxML web servers. Syst. Biol. 57, 758–771. 10.1080/10635150802429642 18853362

[B54] Sun X. Wang G.-L. (2011). Genome-wide identification, characterization and phylogenetic analysis of the rice LRR-Kinases. PLoS ONE 6:e16079. 10.1371/journal.pone.0016079 21408199 PMC3050792

[B55] Sun Y. Han Z. Tang J. Hu Z. Chai C. Zhou B.. (2013a). Structure reveals that BAK1 as a co-receptor recognizes the BRI1-bound brassinolide. Cell Res. 23, 1326–1329. 10.1038/cr.2013.131 24126715 PMC3817550

[B56] Sun Y. Li L. Macho A. P. Han Z. Hu Z. Zipfel C.. (2013b). Structural basis for flg22-induced activation of the *Arabidopsis* FLS2-BAK1 immune complex. Science 342, 624–628. 10.1126/science.1243825 24114786

[B57] Tamura K. Stecher G. Peterson D. Filipski A. Kumar S. (2013). MEGA6: Molecular evolutionary genetics analysis version 6.0. Mol. Biol. Evol. 30, 2725–2729. 10.1093/molbev/mst197 24132122 PMC3840312

[B58] Torii K. U. (2004). Leucine-rich repeat receptor kinases in plants: structure, function, and signal transduction pathways. Int. Rev. Cytol. 234, 1–46. 10.1016/s0074-7696(04)34001-5 15066372

[B59] Wang J. H. Kucukoglu M. Zhang L. B. Chen P. Decker D. Nilsson O.. (2013). The *Arabidopsis* LRR-RLK, *PXC1*, is a regulator of secondary wall formation correlated with the TDIF-PXY/TDR-WOX4 signaling pathway. BMC Plant Biol. 13:94. 10.1186/1471-2229-13-94 23815750 PMC3716795

[B60] Wei Z. Wang J. Yang S. Song Y. (2015). Identification and expression analysis of the *LRR-RLK* gene family in tomato (*Solanum lycopersicum*) Heinz 1706. Genome 58, 121–134. 10.1139/gen-2015-0035 26207619

[B61] Xia X. (2013). DAMBE5: A comprehensive software package for data analysis in molecular biology and evolution. Mol. Biol. Evol. 30, 1720–1728. 10.1093/molbev/mst064 23564938 PMC3684854

[B62] Xu B. Yang Z. H. (2013). pamlX: a graphical user interface for PAML. Mol. Biol. Evol. 30, 2723–2724. 10.1093/molbev/mst179 24105918

[B63] Yang Z. Bielawski J. P. (2000). Statistical methods for detecting molecular adaptation. Trends Ecol. Evol. 15, 496–503. 10.1016/S0169-5347(00)01994-7 11114436 PMC7134603

[B64] Zan Y. Ji Y. Zhang Y. Yang S. Song Y. Wang J. (2013). Genome-wide identification, characterization and expression analysis of populus leucine-rich repeat receptor-like protein kinase genes. BMC Genomics 14:318. 10.1186/1471-2164-14-318 23663326 PMC3682895

[B65] Zhang X. S. Choi J. H. Heinz J. Chetty C. S. (2006). Domain-specific positive selection contributes to the evolution of *Arabidopsis* leucine-rich repeat receptor-like kinase (LRR RLK) genes. J. Mol. Evol. 63, 612–621. 10.1007/s00239-005-0187-z 17031460

[B66] Zhou F. Guo Y. Qiu L.-J. (2016). Genome-wide identification and evolutionary analysis of leucine-rich repeat receptor-like protein kinase genes in soybean. BMC Plant Biol. 16:58. 10.1186/s12870-016-0744-1 26935840 PMC4776374

