# Supplementary figures and images for: Duplication and Divergence of Leucine-Rich Repeat Receptor-Like Protein Kinase (LRR-RLK) Genes in Basal Angiosperm Amborella trichopoda
Source: Front Plant Sci. 2016 Dec 23;7:1952. doi: 10.3389/fpls.2016.01952 (PMC5179525; doi:10.3389/fpls.2016.01952)

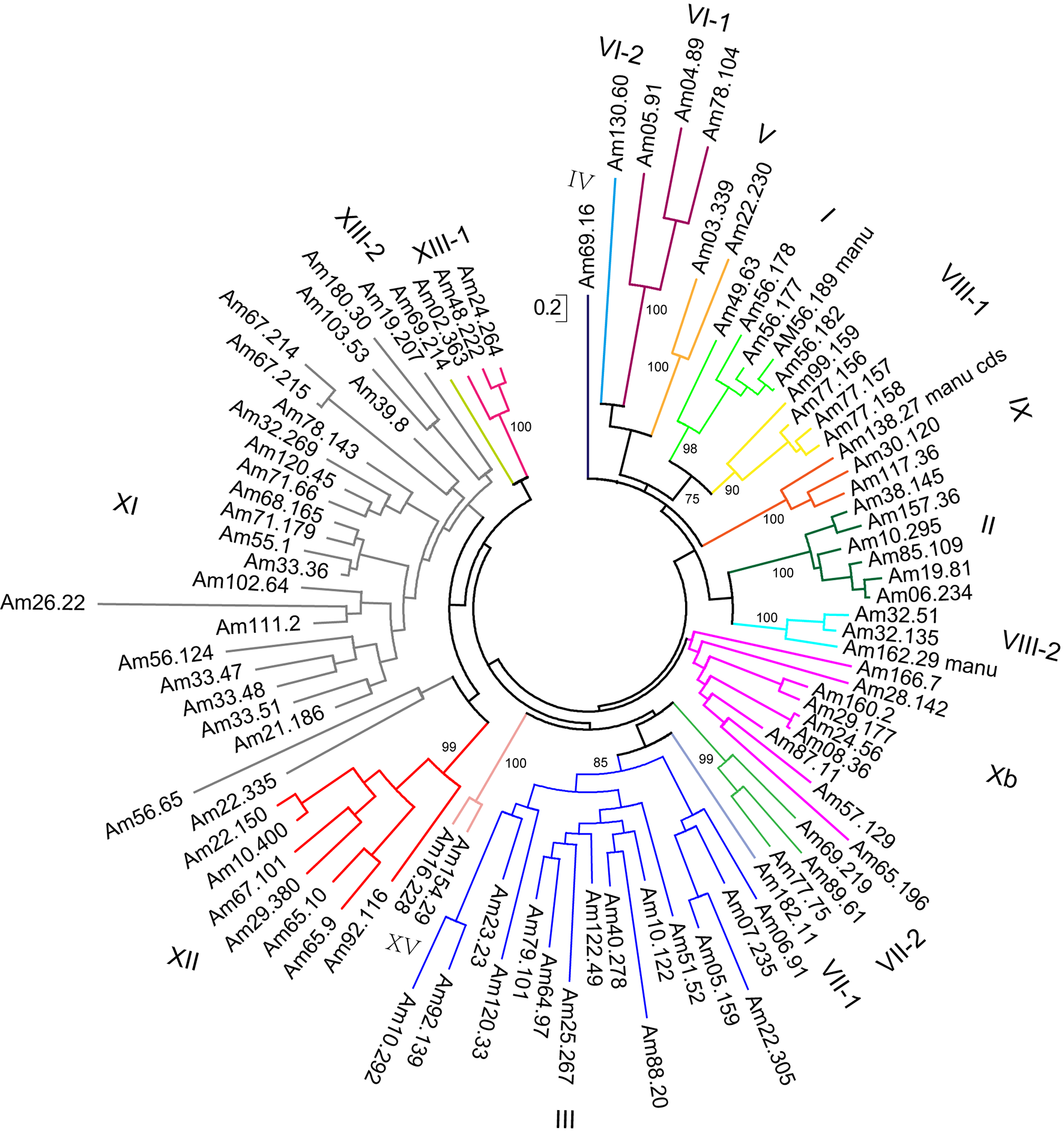

Supplement: Supplemental Figure 1 — Phylogeny of LRR-RLK genes in Amborella trichopoda. This phylogenetic tree based on kinase domain sequence was constructed by the Maximum Likelihood method. [file Image1.TIF]

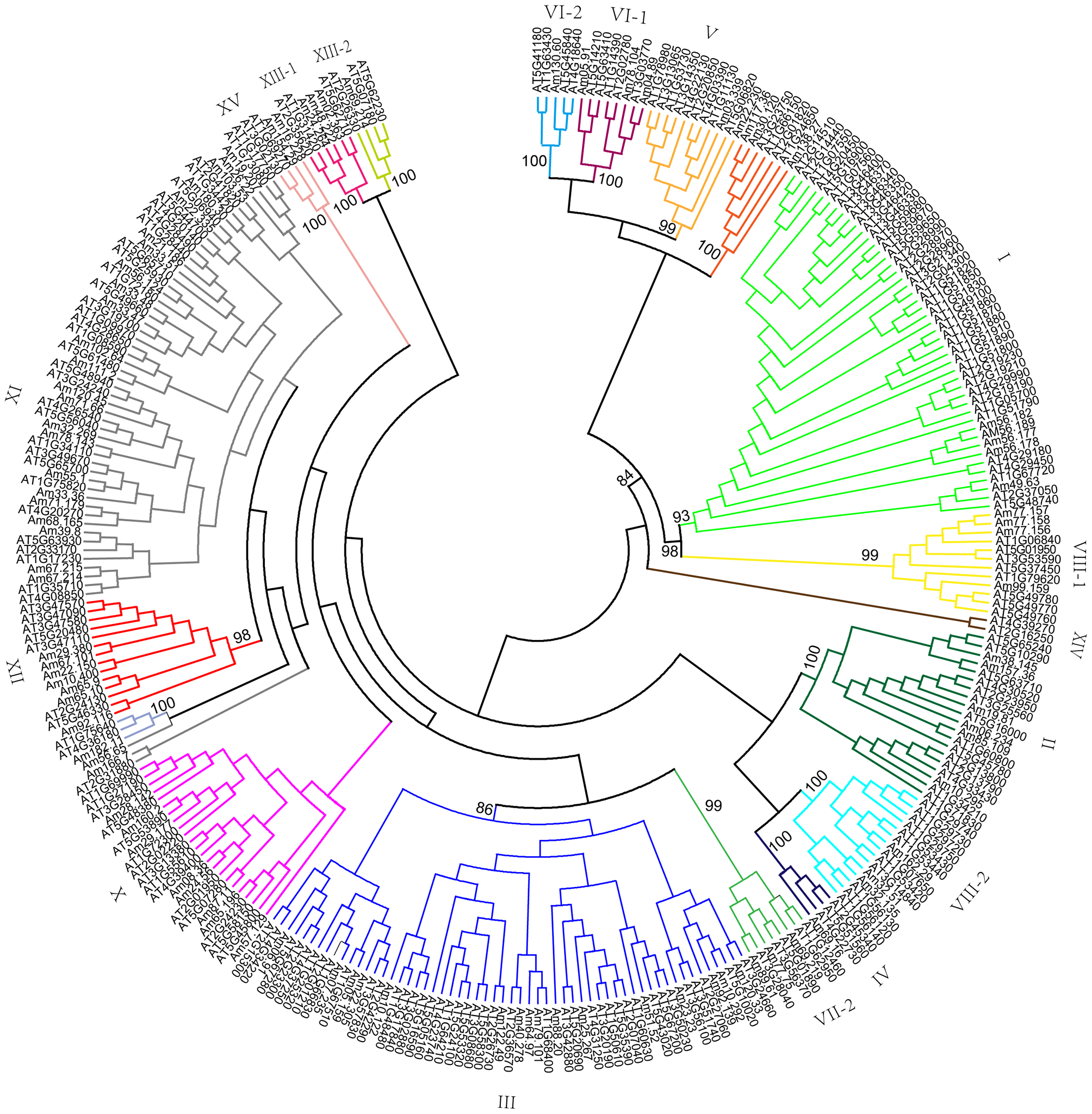

Supplement: Supplemental Figure 2 — Phylogeny of LRR-RLK genes in Amborella trichopoda and Arabidopsis thaliana. This phylogenetic tree based on kinase domain sequence was constructed by the Maximum Likelihood method. [file Image2.TIF]
