# Supplementary material for: Duplication and Divergence of Leucine-Rich Repeat Receptor-Like Protein Kinase (LRR-RLK) Genes in Basal Angiosperm Amborella trichopoda
Source: Front Plant Sci. 2016 Dec 23;7:1952. doi: 10.3389/fpls.2016.01952 (PMC5179525; doi:10.3389/fpls.2016.01952)
Supplement: Supplemental Table 1 — The full name of 94 LRR-RLK proteins identified in the Amborella trichopoda genome. [file Table1.PDF]

Supplemental Table 1: The full name of 94 LRR-RLK proteins identified in the *Amborella trichopoda* genome.

Abbreviation	Full ID
AM56.189	evm_27.TU.AmTr_v1.0_scaffold00056.189
Am56.182	evm_27.TU.AmTr_v1.0_scaffold00056.182
Am56.177	evm_27.TU.AmTr_v1.0_scaffold00056.177
Am56.178	evm_27.TU.AmTr_v1.0_scaffold00056.178
Am49.63	evm_27.TU.AmTr_v1.0_scaffold00049.63
Am77.157	evm_27.TU.AmTr_v1.0_scaffold00077.157
Am77.158	evm_27.TU.AmTr_v1.0_scaffold00077.158
Am77.156	evm_27.TU.AmTr_v1.0_scaffold00077.156
Am99.159	evm_27.TU.AmTr_v1.0_scaffold00099.159
Am04.89	evm_27.TU.AmTr_v1.0_scaffold00004.89
Am78.104	evm_27.TU.AmTr_v1.0_scaffold00078.104
Am05.91	evm_27.TU.AmTr_v1.0_scaffold00005.91
Am130.60	evm_27.TU.AmTr_v1.0_scaffold00130.60
Am22.230	evm_27.TU.AmTr_v1.0_scaffold00022.230
Am03.339	evm_27.TU.AmTr_v1.0_scaffold00003.339
Am69.16	evm_27.TU.AmTr_v1.0_scaffold00069.16
Am30.120	evm_27.TU.AmTr_v1.0_scaffold00030.120
Am117.36	evm_27.TU.AmTr_v1.0_scaffold00117.36
Am138.27	evm_27.TU.AmTr_v1.0_scaffold00138.27
Am19.81	evm_27.TU.AmTr_v1.0_scaffold00019.81
Am06.234	evm_27.TU.AmTr_v1.0_scaffold00006.234
Am85.109	evm_27.TU.AmTr_v1.0_scaffold00085.109
Am10.295	evm_27.TU.AmTr_v1.0_scaffold00010.295
Am38.145	evm_27.TU.AmTr_v1.0_scaffold00038.145
Am157.36	evm_27.TU.AmTr_v1.0_scaffold00157.36
Am32.135	evm_27.TU.AmTr_v1.0_scaffold00032.135
Am32.51	evm_27.TU.AmTr_v1.0_scaffold00032.51
Am162.29	evm_27.TU.AmTr_v1.0_scaffold00162.29
Am57.129	evm_27.TU.AmTr_v1.0_scaffold00057.129
Am65.196	evm_27.TU.AmTr_v1.0_scaffold00065.196
Am87.11	evm_27.TU.AmTr_v1.0_scaffold00087.11
Am24.56	evm_27.TU.AmTr_v1.0_scaffold00024.56
Am08.36	evm_27.TU.AmTr_v1.0_scaffold00008.36
Am160.2	evm_27.TU.AmTr_v1.0_scaffold00160.2
Am29.177	evm_27.TU.AmTr_v1.0_scaffold00029.177
Am28.142	evm_27.TU.AmTr_v1.0_scaffold00028.142
Am166.7	evm_27.TU.AmTr_v1.0_scaffold00166.7
Am64.97	evm_27.TU.AmTr_v1.0_scaffold00064.97

Am79.101	evm_27.TU.AmTr_v1.0_scaffold00079.101
Am25.267	evm_27.TU.AmTr_v1.0_scaffold00025.267
Am122.49	evm_27.TU.AmTr_v1.0_scaffold00122.49
Am88.20	evm_27.TU.AmTr_v1.0_scaffold00088.20
Am40.278	evm_27.TU.AmTr_v1.0_scaffold00040.278
Am10.122	evm_27.TU.AmTr_v1.0_scaffold00010.122
Am51.52	evm_27.TU.AmTr_v1.0_scaffold00051.52
Am92.139	evm_27.TU.AmTr_v1.0_scaffold00092.139
Am10.292	evm_27.TU.AmTr_v1.0_scaffold00010.292
Am23.23	evm_27.TU.AmTr_v1.0_scaffold00023.23
Am120.33	evm_27.TU.AmTr_v1.0_scaffold00120.33
Am22.305	evm_27.TU.AmTr_v1.0_scaffold00022.305
Am05.159	evm_27.TU.AmTr_v1.0_scaffold00005.159
Am07.235	evm_27.TU.AmTr_v1.0_scaffold00007.235
Am06.91	evm_27.TU.AmTr_v1.0_scaffold00006.91
Am182.11	evm_27.TU.AmTr_v1.0_scaffold00182.11
Am89.61	evm_27.TU.AmTr_v1.0_scaffold00089.61
Am77.75	evm_27.TU.AmTr_v1.0_scaffold00077.75
Am69.219	evm_27.TU.AmTr_v1.0_scaffold00069.219
Am154.29	evm_27.TU.AmTr_v1.0_scaffold00154.29
Am16.228	evm_27.TU.AmTr_v1.0_scaffold00016.228
Am33.47	evm_27.TU.AmTr_v1.0_scaffold00033.47
Am33.48	evm_27.TU.AmTr_v1.0_scaffold00033.48
Am56.124	evm_27.TU.AmTr_v1.0_scaffold00056.124
Am33.51	evm_27.TU.AmTr_v1.0_scaffold00033.51
Am21.186	evm_27.TU.AmTr_v1.0_scaffold00021.186
Am26.22	evm_27.TU.AmTr_v1.0_scaffold00026.22
Am111.2	evm_27.TU.AmTr_v1.0_scaffold00111.2
Am102.64	evm_27.TU.AmTr_v1.0_scaffold00102.64
Am68.165	evm_27.TU.AmTr_v1.0_scaffold00068.165
Am71.179	evm_27.TU.AmTr_v1.0_scaffold00071.179
Am55.1	evm_27.TU.AmTr_v1.0_scaffold00055.1
Am33.36	evm_27.TU.AmTr_v1.0_scaffold00033.36
Am120.45	evm_27.TU.AmTr_v1.0_scaffold00120.45
Am71.66	evm_27.TU.AmTr_v1.0_scaffold00071.66
Am32.269	evm_27.TU.AmTr_v1.0_scaffold00032.269
Am78.143	evm_27.TU.AmTr_v1.0_scaffold00078.143
Am67.214	evm_27.TU.AmTr_v1.0_scaffold00067.214
Am67.215	evm_27.TU.AmTr_v1.0_scaffold00067.215
Am39.8	evm_27.TU.AmTr_v1.0_scaffold00039.8
Am180.30	evm_27.TU.AmTr_v1.0_scaffold00180.30
Am103.53	evm_27.TU.AmTr_v1.0_scaffold00103.53
Am19.207	evm_27.TU.AmTr_v1.0_scaffold00019.207

Am29.380	evm_27.TU.AmTr_v1.0_scaffold00029.380
Am67.101	evm_27.TU.AmTr_v1.0_scaffold00067.101
Am10.400	evm_27.TU.AmTr_v1.0_scaffold00010.400
Am22.150	evm_27.TU.AmTr_v1.0_scaffold00022.150
Am65.9	evm_27.TU.AmTr_v1.0_scaffold00065.9
Am65.10	evm_27.TU.AmTr_v1.0_scaffold00065.10
Am92.116	evm_27.TU.AmTr_v1.0_scaffold00092.116
Am56.65	evm_27.TU.AmTr_v1.0_scaffold00056.65
Am22.335	evm_27.TU.AmTr_v1.0_scaffold00022.335
Am69.214	evm_27.TU.AmTr_v1.0_scaffold00069.214
Am48.222	evm_27.TU.AmTr_v1.0_scaffold00048.222
Am24.264	evm_27.TU.AmTr_v1.0_scaffold00024.264
Am02.363	evm_27.TU.AmTr_v1.0_scaffold00002.363
